# Reading Russian poetry: An expert–novice study

**DOI:** 10.16910/jemr.13.3.7

**Published:** 2022-05-16

**Authors:** Danil Fokin, Stefan Blohm, Elena Riekhakaynen

**Affiliations:** University of Warsaw, Poland; Saint-Petersburg State University, Russia; Radboud University, Nijmegen, Netherlands; Max Planck Institute for Empirical Aesthetics, Frankfurt am Main, Germany

**Keywords:** Eye tracking, reading, poetry, art reception, individual differences, expert–novice, expertise

## Abstract

Studying the role of expertise in poetry reading, we hypothesized that poets’ expert knowledge comprises genre-appropriate reading- and comprehension strategies that are re-flected in distinct patterns of reading behavior.

We recorded eye movements while two groups of native speakers (n=10 each) read selected Russian poetry: an expert group of professional poets who read poetry daily, and a control group of novices who read poetry less than once a month. We conducted mixed-effects re-gression analyses to test for effects of group on first-fixation durations, first-pass gaze du-rations, and total reading times per word while controlling for lexical- and text variables.

First-fixation durations exclusively reflected lexical features, and total reading times re-flected both lexical- and text variables; only first-pass gaze durations were additionally mod-ulated by readers’ level of expertise. Whereas gaze durations of novice readers became faster as they progressed through the poems, and differed between line-final words and non-final ones, poets retained a steady pace of first-pass reading throughout the poems and within verse lines. Additionally, poets’ gaze durations were less sensitive to word length.

We conclude that readers’ level of expertise modulates the way they read poetry. Our find-ings support theories of literary comprehension that assume distinct processing modes which emerge from prior experience with literary texts.

## Introduction

The reception and appreciation of art crucially depends on the expertise of the recipient (23). Experts possess knowledge and skills that allow them to approach works of art in ways that are unavailable to novices. Prior research in the domain of verbal art has confirmed that poets – experts in the art of poetry – possess superior relevant skills and pursue different strategic approaches to poetry than novices (24; 34; 38).

However, it remains unclear in how far the process of poetry reading is affected by readers’ level of expertise. Here, we report results of an expert–novice study of poetry reading in Russian, a language that is underrepresented in research into poetry reception. Word-level analyses of selected eye-movement measures that tap into distinct processing stages during reading confirm the hypothesis that expert knowledge affects poetry reading.

Prior research has established that reading experience and exposure to print material improves reading skills and reading efficiency in adults (14; 37), affecting not only high-level comprehension processes like inferencing (25) but also low-level processes like word recognition ( 2; 7). For instance, skilled readers with a high degree of prior print exposure have shorter gaze durations, skip words more frequently, and are less susceptible to the influence of low-level word characteristics like word length and -frequency (7; 9; 10; 14). Readers with high exposure to specific text genres (e.g., academic prose, lyrical poetry) are likely to acquire genre-specific expert knowledge beyond the general benefits of print exposure for text reading and comprehension. Sufficient experience with literary texts, for instance, leads to the emergence of distinct processing- and comprehension strategies for literary vs. non-literary texts (4; 15; 42) and for different literary genres (6; Peskin, 2007). Literary text reading is usually slower than non-literary reading, and it results in improved verbatim memory (15) but less accurate memory for situational information (42). Prior results further indicate that poetry-appropriate processing strategies modulate readers’ attentional state even prior to reading (5), affect readers’ processing routines and interpretive operations during reading (6; 11, Peskin, 2007), and result in improved verbatim memory after reading (15; 24). Poetry reading appears to be more careful than prose reading, since it is characterized by reduced reading speed, shorter progressive saccades and less frequent word skipping (6), as well as increased total word reading times and a greater tendency to re-read earlier sections of the text (11). Since such poetry-appropriate strategies depend on prior experience and practice, it seems reasonable to assume that they are particularly pronounced, or nuanced, in poets. Here, we examine whether inspection-time measures of reading behavior are indicative of such expertise-dependent reading strategies.

The expert knowledge of poets has been examined in prior research. Early findings revealed poets’ special abilities with respect to formal (rhyming), lexical, and semantic (verbal imagery and metaphor) aspects of poetry, as well as good verbatim memory for poetic material (38). More recent investigations have refined our knowledge about the psychology of poetic expertise, e.g., poets’ implicit knowledge of rhyming (8), or expertise-dependent memory effects (24). Corpus-based research has revealed expertise- and skill-dependent differences in the poetic practices of professional vs. amateur poets, e.g., in terms of lexical choice and the treatment of poetic form (19). Whether and how poets’ expertise affects the process of reading poetry remains unclear, though. The present study addresses this question by comparing eye movements recorded while poets (experts) and novices silently read selected poetry; its focus on inter-individual differences and on Russian poetry broadens the scope of contemporary eye-tracking research into poetry reading (6; 11; 13; 17; 21; 26; 28; 30; 32).

The primary aim of the current study was to assess whether and how expert knowledge affects poetry reading. To this end, we recorded eye movements while two age-matched groups of professional poets (i.e., experts) and novices read selected Russian poetry. Expecting that expertise-dependent reading- and comprehension strategies would lead to systematic between-group differences in reading behavior, we conducted word-level analyses of the reading process. First-fixation duration was selected as an index of early stages of word processing during reading, e.g., word recognition. We expected that this measure would be modulated by lexical properties of the words, e.g., word length and -frequency. Finding this measure sensitive to readers’ level of expertise (poets vs. novices) would support the hypothesis that poets’ reading strategy for poetry affects early stages of the reading process. Gaze duration was selected as an index of first-pass reading, which additionally reflects later and more controlled stages of comprehension, e.g., the semantic integration of a word into the previous discourse. We expected this measure to be sensitive to the level of expertise, reflecting that poets’ reading strategy for poetry emphasizes different aspects of the text, e.g., its rhythmic structure or its semantic polyvalence. Total reading time was selected as the most general index of the reading process that additionally reflects the re-reading(s) of a word, either because readers regressed locally within the text or because they re-read the entire poem. While we expected to observe expertise-dependent differences in this measure, we were cautious to base strong conclusions on potential differences in total reading times, since this measure may partly reflect strategic re-reading due to task requirements.

## Methods

### Participants

We recruited a sample (*n* = 11) of professional poets (laureates, nominees or winners of poetry awards) by personal appeal (five females, *M*age = 38.0, *SD*age = 12.1, age range: 25–60 years). All experts engaged in literary activities professionally and reported to read poetry daily. Data from one expert had to be discarded due to a technical error during eye-movement recording so that data from ten experts entered the analysis; one expert contributed only three trials since one trial was excluded due to signal loss during recording.

We further recruited an age-matched (unpaired t-test: *t* (18) = 0.59, *p* = .563) control group of novice readers who reported to read poetry less than once a month. Data from two novices had to be discarded due to poor calibration and a technical error during eye-movement recording; data from ten novices entered the analysis (seven females, *M*age = 34.7, *SD*age = 12.9, age range: 19–62 years).

All participants were native speakers of Russian, had normal or corrected-to-normal vision, and were naïve to the purpose of the research. All participants gave written informed consent prior to the experiment.

### Stimuli and Design

We selected four Russian poems from the late 20th century (see Table 1). This is a particularly influential era in recent Russian poetics in which poets built on the achievements of earlier centuries and introduced novel trends into poetry (22).

All four texts were presented to each participant. We expected that expertise-induced differences in reading behavior should become apparent as between-group differences.

**Table 1.** Stimulus Texts.

Poem (author)	Lines	Words	Log-freq. *M* (*SD*)	Word length *M* (*SD*)	Orth. neigh.
*Mozart*^1^ (Shwartz)	17	69	2.2 (1.3)	5.4 (2.8)	5.6
*Kambala*^2^ (Shwartz)	17	66	2.1 (1.5)	4.8 (2.8)	5.6
*Kabel*^3^ (Tsvetkov)	16	65	1.7 (1.4)	5.6 (2.8)	6.3
*Polinya*^4^ (Tsvetkov)	16	60	2.0 (1.4)	5.5 (2.8)	4.5

^1^ https://pub.wikireading.ru/11503/ (accessed 05.01.2022). ^2^ https://pub.wikireading.ru/11493 (accessed 05.01.2022). ^3^ http://www.vavilon.ru/texts/tsvetkov1-1.html (accessed 05.01.2022). ^4^ http://www.vavilon.ru/texts/tsvetkov1-1.html (accessed 05.01.2022).

### Procedure

Prior to the experiment, participants gave written informed consent to volunteer as participant in the study. In a brief questionnaire, they supplied demographic data (age, gender), indicated whether they engaged with poetry professionally, and reported how frequently they read poetry.

The main experiment took about 30-45 minutes and was conducted in a well-lit and sound-attenuated room. Participants were instructed that they would read poems written by Russian authors after World War II, and that they would be asked to respond to some questions about these texts after reading; instructions asked participants to read the poems "attentively in convenient tempo".

The experiment began with a practice trial to familiarize participants with the reading situation and the subsequent tasks; for practice we presented the first stanza of Mikhail Aizenberg’s poem “The soot is white no matter how blackened…” («Сажа бела, сколько б не очерняли…» (21 words, four sentences, https://znamlit.ru/publication.php?id=5797). Following practice, participants read all four poems in randomized order while their eye movements were recorded.

Each trial began with a standard 9-point calibration and validation procedure to ensure a spatial resolution error of less than 0.5° of visual angle. Text presentation was triggered when participants fixated a black dot (16 points) conveniently located to the left of where the first word of the text would appear. Texts were left-aligned and displayed in a 25-point Times New Roman font with 1.5-line spacing.

Participants were free to read the poems at their own pace and to go back and forth within as often as they wanted without a time limit. After reading a poem, participants pressed the spacebar to proceed to three oral tasks: a free-association task, in which they were required to name any associations they had after reading the text, a keyword-task, in which they were required to name the words of the poem they considered most significant for its interpretation, and a cloze task, in which they were presented with the poem again and required to fill in gaps. i.e., individual words that had been left out. Data from the oral naming tasks are not reported here; the interested reader is referred to (12); results of an unpaired *t*-test of mean accuracy rates indicated that both groups performed equally well in the cloze task (*t*(18) = 0.79, *p* = .438).

### Recording

Participants’ eye movements were sampled at 1000 Hz with a desktop mount EyeLink 1000 Plus eye tracker (SR Research Ltd., Mississauga, Ontario, Canada). Stimulus presentation was controlled by SR Research Experiment Builder (version 2.3.38). Stimuli were presented on a 19-inch LCD monitor with a refresh rate of 60 Hz and a resolution of 1600x1024 pixels. Distance from participants’ eyes to the stimulus monitor was approximately 80 cm. A head-and-chin rest was used to minimize participants’ head movements. Viewing was binocular but only the left eye was recorded. Participants’ responses to oral experiments were recorded with App-Dictaphone (Appliqato Software, Nicosia, Cyprus), using Huawei AMN LX-9 (2019).

### Data analysis

Raw data were checked manually before we applied an automatic cleaning procedure, accepting fixations between 50 ms and 800 ms. Then we extracted interest area reports, using each word as an individual interest area; we analyzed first fixation durations, gaze durations, and total reading times; skipped words were treated as missing observations. The underlying data are provided in the supplementary material (https://osf.io/bzcra/).

We removed outlying values exceeding participant-specific cutoffs (mean + 3 *SD*s) before we analyzed word-level data using linear mixed-effects regression with random effects for participants. Outlier removal resulted in data loss of less than 2% in all cases; remaining observations were distributed evenly across groups although one trial from the poet group had to be discarded (Chi-squared tests for given probabilities: all *χ^2^*(1) < 2.8; all *p*s > .095). We then aimed to fit parsimonious models using a three-step selection procedure that involved both forward- and backward-fitting. Analyses were carried out in JASP (18); the analysis file is available at https://osf.io/nqxhe/.

1. First we fitted a base model that contained fixed main and two-way interaction effects of lexical- and text-related variables, which allowed us to control for differences between words and to approximate how readers navigated through the poems. Lexical variables included *word length* (i.e., the number of letters per word), *log-frequency* (i.e., the log-transformed number of occurrences in the Frequency Dictionary of Modern Russian; 27) as well as *orthographic-neighborhood size* (i.e., the number of transposition- and substitution neighbors retrieved from a lexical database of modern Russian; 1), all of which have been identified as relevant lexical variables in prior studies of eye movements during poetry reading (41, 40); expectably, all lexical variables showed moderately strong correlations (0.3 < *r* < 0.7): length – frequency (*r* = -0.69), length – orth. neighb. (*r* = -0.56), frequency – orth. neighb. (*r* = 0.45). Text-related variables included the serial *text position* of each word (i.e., 1^st^ word, 2^nd^ word, etc.) as well as its *line position* (final vs. non-final), which has been shown to influence reading times in poetry comprehension (11). Including main and interaction effects of text- and line position allowed us to approximate how participants navigated through the poems. Although this is, admittedly, a crude approximation that reduces texts to a linear sequence of words and disregards most of the text structure, e.g., the division of poems into lines and stanzas (3; 11; 30), we refrained from including further structure-related variables, since the available data did not support overly complex models; higher-order interactions were excluded for the same reason. We then eliminated non-significant predictors from the initial base model in a stepwise fashion, using a liberal alpha level of *p* < .1.

2. In a second step, we added the main variables of interest to the (back-fitted) base model, i.e., fixed main and two-way interaction effects of *group* (experts vs. novices). Subsequently, we reduced this extended model in a stepwise fashion again, now using the stricter conventional alpha level *p* < .05.

3. In the final step, we forward-fitted the random-effect structure of the (back-fitted) extended models, testing whether random slopes for main effects improved the fit of the models, as indexed by the AIC; random slopes were tested in the order in which we included fixed-effects terms, i.e., lexical variables > text-related variables > reader variables. Since including random slopes affects the coefficient estimates of the respective fixed-effects terms, we checked whether the resulting models included non-significant (*p* > .05) predictors and removed them if appropriate; non-significant main effects were kept if they were part of higher-order interactions.

## Results

We conducted linear mixed-effects regression analyses of first-fixation durations, gaze durations, and total reading times per word. We report the ANOVA summaries of the final statistical models determined in the model selection procedure. Our primary interest was in main and interaction effects of group, which we assume to reflect expertise-dependent adjustments of reading behavior; these effects are described in detail here. By contrast, main and interaction effects of lexical- and text variables are reported only briefly; we refer the interested reader to the Appendix for post-hoc analyses of the interaction effects.

### First-fixation duration

The final regression model included only the lexical variables word length, word frequency and orthographic neighborhood size as well as their interaction as predictors (see Table 2); there were no effects of group on first-fixation duration.

**Table 2.** First-Fixation Duration: ANOVA Summary.

Effect	*Df*	*F*	*P*
Word length	1, 4757	5.46	.019
Orthographic neighborhood	1, 4757	18.33	< .001
Log-frequency	1, 4757	21.27	< .001
Word length * Orth. neighb.	1, 4757	20.58	< .001
Orth. neighb * Log-frequency	1, 4757	6.02	.014

Note. Model terms were tested with Satterthwaite method; random effects grouping factor: ‘participant’.

The main effect of word length (p = .019) indicated that long words required longer first fixations than short words. The main effect of lexical frequency (p < .001) indicated that, in line with previous results (2; 20; 35), first fixations on high-frequency words were shorter than those on low-frequency words. The main effect of orthographic-neighborhood size (p < .001) indicated that first fixations were longer for words with many competing neighbors than for words with no or only a few neighbors. Both the advantage for high-frequency words and the penalty for long words were moderated by the number of orthographic neighbors. The interaction of word length and orthographic-neighborhood size (p < .001) reflected that the detrimental effect of word length increased with the number of orthographic neighbors; words without orthographic neighbors showed no word-length effect at all (see Table A1 in the Appendix). The interaction of lexical frequency and orthographic-neighborhood size (p = .014) indicated that the facilitation effect for high-frequency words was strongest for words with few orthographic neighbors (~1 word) and decreased with increasing numbers of orthographic competitors; words with large orthographic neighborhoods (~14 words) showed no frequency effect at all (see Table A2 in the Appendix).

### First-pass gaze duration

The final regression model included random effects for participants, and fixed effects of all lexical variables (word length, word frequency, orthographic neighborhood), text-related variables (text- and line position) and group as well as several interactions (see Table 3).

**Table 3.** Gaze duration: ANOVA summary.

Effect	*df*	*F*	*p*
Word length	1, 4734	44.37	< .001
Orthographic neighborhood	1, 4727	12.07	< .001
Group	1, 53	0.17	.678
Log-frequency	1, 47	30.47	< .001
Text position	1, 4740	5.77	.016
Line position	1, 4725	9.39	.002
Word length * Orth. neighb.	1, 4730	5.62	.018
Word length * Group	1, 1332	18.45	< .001
Orth. neighb. * Log-frequency	1, 4732	14.16	< .001
Word length * Text position	1, 4738	15.93	< .001
Group * Text position	1, 4737	4.20	.041
Group * Line position	1, 4734	9.21	.002

Note. Model terms were tested with Satterthwaite method; random effects grouping factor: ‘participant’.

We observed main effects of word length, orthographic-neighborhood size and word frequency (all ps < .001), which indicated that first-pass reading was faster for short words than for long ones, faster for words with small neighborhood sizes than for those with many orthographic competitors, and faster for high-frequency vs. low-frequency words. Main effects of serial text position (p = .016) and of line position (p = .002) reflected that gaze durations were shorter for words occurring later in the poem than for those at the beginning, and for words in line-final position than for words occurring earlier in the line. These main effects of word- and text variables were qualified by several interactions.

The interaction of word length and orthographic neighborhood (p = .018) reflected that greater numbers of orthographic neighbors increased the detrimental effect of word length (see Table A3 in the Appendix), whereas the interaction of orthographic neighborhood and log-frequency (p < .001) indicated that greater numbers of orthographic neighbors reduced the facilitative effect of high frequency (see Table A4 in the Appendix). The interaction of word length and text position (p < .001) reflected that the word-length effect was strongest for words that occurred early in the text and decreased as readers progressed through the poems (see Table A5 in the Appendix).

**Figure 1.**
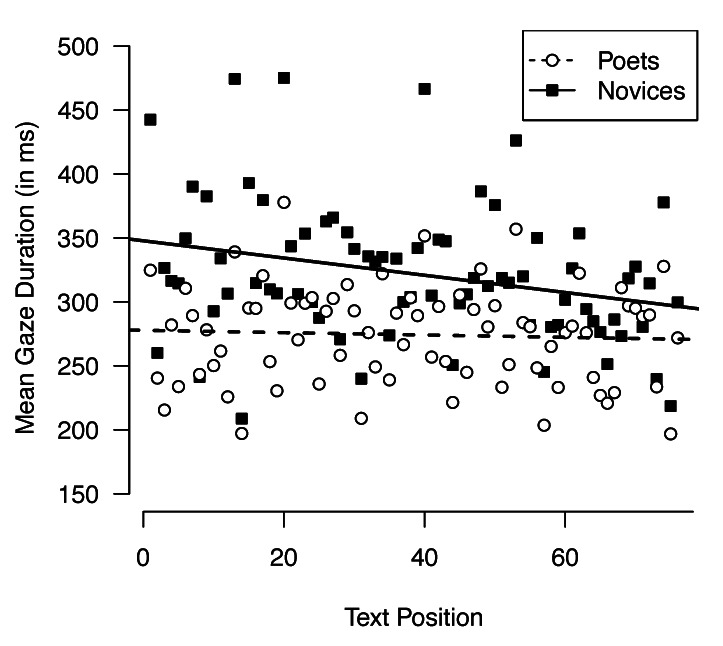
Mean gaze durations during poetry reading as a function of serial text position (1^st^ word, 2^nd^ word, …, n^th^ word) and level of expertise (poets vs. novices).

Crucially, first-pass gaze durations revealed distinct patterns in the two groups of readers. There was no main effect of group (p = .678), but we observed interactions of group and word length (p < .001), group and text position (p = .041), as well as group and line position (p = .002). While gaze durations of both groups were affected by word length (longer words took longer to read), this effect was more pronounced in novice readers (B = 17 (±2), CI_95%_ = [14, 20], p < .001) than in poets (B = 9 (±2), CI_95%_ = [6, 12], p < .001), i.e., poets were less sensitive to this low-level lexical variable. Moreover, gaze durations of novice readers became faster as they progressed through the poems (B = -0.5 (±0.1), CI_95%_ = [-0.8, -0.2], p = .001) but first-pass reading of poets showed no such tendency (B = -0.1 (±0.1), CI_95%_= [-0.4, 0.2], p = .548); see Figure 1.

**Figure 2.**
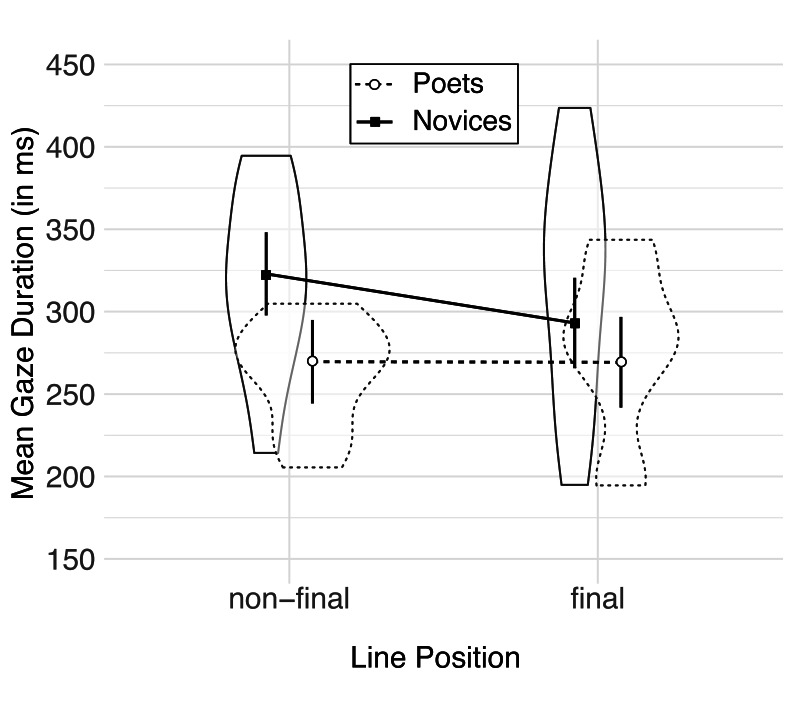
Mean gaze duration per word during poetry reading as a function of line position (non-final vs. final) and level of expertise (poets vs. novices).

Similarly, gaze durations of novice readers were sensitive to the position of a word within the verse line (non-final vs. final) such that line-final words were read faster than non-final ones (M_final_ = 293, M_non-final_ = 323, z = 4.34, p < .001), whereas poets’ gaze durations were unaffected by the line position (M_final_ = 269, M_non-final_ = 269, z = 0.05, p = .959); see Figure 2.

### Total reading times

The final model included fixed effects of all lexical variables (word length, word frequency, orthographic neighborhood) and text-related variables (text- and line position) as well as several interactions. Contrary to our expectations, there were no effects of group on total reading times (see Table 4).

**Table 4.** Total reading time: ANOVA summary.

Effect	*df*	*F*	*P*
Word length	1, 4722	58.97	< .001
Orthographic neighborhood	1, 4722	20.73	< .001
Log-frequency	1, 4722	149.37	< .001
Text position	1, 4722	5.75	.017
Line position	1, 131	1.52	.220
Word length * Orth. neighb.	1, 4722	18.67	< .001
Orth. neighb. * Log-frequency	1, 4722	6.26	.012
Word length * Text position	1, 4722	23.74	< .001
Word length * Line position	1, 4724	5.38	.020

Note. Model terms were tested with Satterthwaite method; random effects grouping factor: ‘participant’.

Main effects of word length (p < .001), orthographic neighborhood (p < .001), and lexical frequency (p < .001) indicated that greater word length and greater numbers of orthographic neighbors increased total reading times whereas greater word frequency reduced reading times. Additionally, total reading times decreased as readers progressed through the poems (main effect of text position, p = .017). These main effects were qualified by several interactions.

The interaction of word length and orthographic neighborhood (p < .001) reflected that greater numbers of orthographic neighbors increased the detrimental effect of word length (see Table A6 in the Appendix). The interaction of word frequency and orthographic neighborhood (p = .012) indicated that greater numbers of orthographic neighbors reduced the facilitation effect observed for high-frequency words (see Table A7 in the Appendix). The interaction of word length and text position (p < .001) reflected that the word-length effect was strongest for words that occurred early in the text and decreased as readers progressed through the poems (see Table A8 in the Appendix). Finally, the interaction of word length and line position (p = .020) revealed that readers spent less time on line-final words than on non-final ones (see Table A9 in the Appendix).

## Discussion

We examined whether expert knowledge affects poetry reading. Selected Russian poems were presented to two groups of native speakers, professional poets (experts) and an age-matched sample of readers who rarely read poetry (novices). Assuming that frequent poetry reading and expert knowledge lead to the emergence of pronounced genre-appropriate reading- and comprehension strategies, we expected to observe distinct eye-movement patterns in expert- and novice readers. We examined indices of early word processing (first-fixation durations), of first-pass reading (gaze durations), and of the entire reading process, including re-reading (total reading times). We employed linear mixed-effects regression to analyze reading times per word, controlling for major lexical variables (word length, word frequency and orthographic neighborhood) as well as for the serial text position of words and their position within the verse line (non-final vs. final).

First-fixation durations were sensitive to lexical variables but unaffected by text- or line position. Crucially, we observed no systematic differences between poets and novices and thus failed to obtain evidence that early word processing during poetry reading is subject to the top-down control of poets’ genre-specific reading strategy. The lexical effects are largely consistent with prior evidence, replicating the well-established effect of word frequency (35); However, the word-length penalty we observed is at odds with prior results (11). The main and interaction effects of orthographic-neighborhood size can be accounted for in terms of lexical competition between similar words during reading.

Confirming the hypothesis of expertise-dependent reading behavior, first-pass gaze durations were not only modulated by lexical variables but further exhibited distinct patterns in poets and novices. For one, gaze durations of novice readers became shorter while progressing through the poems, whereas professional poets showed no such trend, i.e., first-pass reading in novice readers got faster but poets retained the same pace throughout. Since the pattern observed for novices is consistent with prior evidence that word reading times become faster as text reading progresses (e.g., 39), it is the steady pace observed in poets which is unusual and which presumably forms part of their poetry-appropriate reading strategy. Similarly, poets’ gaze durations were insensitive to the line position of words, but novice readers read line-final words faster than non-final ones. Thus, the reading strategy of poets seems to assign equal importance to all words, both within the entire text and the individual verse line. Notably, the finding that novice readers read line-final words faster than non-final ones is at odds with the results reported by Fechino and colleagues (11), who observed longer reading times for line-final words across inspection-time measures of early and late processing during poetry reading. However, it seems to align with earlier reports of rhyme-induced facilitation during poetry reading (e.g., 16; 29; 31). The source of this discrepancy is not clear at present but it might reflect differences in the end-rhyme schemes of the stimulus texts, which affect the predictability of line-final words.

We further observed an interaction effect of group and word length such that the effect of word length on first-pass gaze durations (longer reading times for longer words) was less pronounced in professional poets than in novice readers. We note that this result resembles similar findings obtained in prior research into literary reading: Analyzing gaze durations of readers reading Dutch short stories, Eekhof and colleagues (9) observed that greater levels of previous print exposure and greater degrees of absorption during reading were associated with decreased sensitivity to word length (and other lexical features). However, it is unclear whether the effect observed in the present study also reflects poets’ immersive reading mode due to their extensive experience with poetry, or whether it merely reflects their general reading efficiency due to a generally high level of print exposure (cf. 7; 14). Future investigations aiming to re-assess the relation between readers’ genre-specific expertise and their sensitivity to lexical variables during reading should control for participants’ level of prior print exposure, e.g., by means of an author recognition test (37), and preferably match groups in terms of this reader variable.

Total reading times showed effects of lexical- and text variables which are consistent with prior evidence and with the effects we observed on indices of earlier (word) processing; the main and interaction effects of orthographic-neighborhood size can be accounted for in terms of lexical competition during reading. The observed effect of text position on total reading times (readers become faster as they progressed through the poems) replicates earlier evidence from poetry reading in German (3; 30). However, we failed to obtain evidence that total reading times during poetry reading differ between experts and novices.

Taken together, our results confirm that expert knowledge affects poetry reading. While we failed to obtain evidence that these reading strategies modulate early word processing during reading (as indexed by first-fixation durations) or late processing (indexed by total reading times), our results identify gaze durations as indices of expertise-dependent reading behavior. We observed the typical text-reading pattern in novices, whose gaze durations became faster as they progressed through the poems. Poets, by contrast, retained a steady pace throughout the poems and within verse lines. These reading pattern map onto proposed reading stances for literary texts (e.g., 17; 36), i.e., aesthetic reading (poets) vs. efferent/immersive reading characteristic of prose reading (novices), and it might reflect that poets aim to read without bias and expect that all words might be of significance. This idea of unbiased reading in experts would account both for poets’ steady pace throughout the texts and for their insensitivity to the distinction between line-final words and non-final ones. The latter, however, might also reflect differences in the sensitivity to rhyme between novices, who might have more traditional conceptions of poetry and stronger rhyme expectations, and poets, who are presumably more familiar with and more inclined towards modern unrhymed poetry.

We note that the depth of our analyses was constrained by the amount of available data, which, in turn, was partly determined by the limited availability of professional poets as participants. Hence, the data at hand did not support complex statistical models including, for instance, more text-structural variables that have been shown to affect poetry reading (3; 30). To better assess whether poetic structure (e.g., stanza form or systematic rhyme) differentially affects poetry reading in expert- and novice readers, care should be taken in future investigations that each reader is presented with a sufficient number of texts. Still, the present results provide initial evidence that experts and novices approach poetry differently, and thus identify readers’ level of expertise as a relevant variable whose influence on the reception of verbal art deserves further investigation. While many models of literary comprehension assume distinct modes of processing and comprehending literary texts (17; 36; 43), our results indicate that – in line with widespread assumptions about art reception in other aesthetic domains (23; 33) – such processing strategies for verbal art are co-determined by recipients’ level of expertise.

### Ethics and Conflict of Interest

The author(s) declare(s) that the contents of the article are in agreement with the ethics described in http://biblio.unibe.ch/portale/elibrary/BOP/jemr/ethics.html and that there is no conflict of interest regarding the publication of this paper.

### Acknowledgements

We thank two anonymous reviewers for providing helpful comments and suggestions on a previous version of the article, and our participants who volunteered to participate in the experiment despite of the pandemic-related restrictions.

This project was enabled by a Radboud Excellence fellowship from Radboud University in Nijmegen, Netherlands. Experiments were conducted on the basis of the Laboratory for cognitive studies, St.Petersburg State University.

## Appendix

We report post-hoc tests of significant interaction effects involving only lexical- and/or text variables. Interactions were resolved by calculating conditional slopes of lexical effects at each level of categorical variables or at fixed values of scaled variables, respectively. Tables present slope estimates, standard errors, and 95% confidence intervals.

**First-fixation durations**

Table A1 displays the interaction effect of word length and orthographic-neighborhood size on first-fixation durations per word. Increasing neighborhood size amplified the word-length effect (long words required longer fixations than short words); only when orthographic neighborhoods were very small (~1 word) did increasing word length tend to reduce fixation durations.

**Table A1.** First-fixation duration: Interaction of word length and orthographic-neighborhood size.

			95% *CI*	
Orthographic neighborhood	Word length (slope)	*SE*	Lower	Upper
1	-2	1	-3	0
8	2	1	1	4
14	6	1	3	9

Table A2 displays the interaction effect of lexical frequency and orthographic-neighborhood size on first-fixation durations per word.

**Table A2.** First-fixation duration: Interaction of lexical frequency and orthographic-neighborhood size.

			95% *CI*	
Orthographic neighborhood	Log-frequency (slope)	*SE*	Lower	Upper
1	-8	2	-12	-5
8	-5	1	-8	-3
14	-2	2	-6	1

Increasing neighborhood size reduced the effect of lexical frequency (high-frequency words required shorter fixations than low-frequency words); words with large orthographic neighborhoods (~14 words) showed no effect of frequency.

**First-pass gaze durations**

Table A3 displays the interaction effect of word length and orthographic-neighborhood size on first-pass gaze durations per word. Increasing neighborhood size amplified the word-length effect (long words took longer to read than short words).

**Table A3.** Gaze duration: Interaction of word length and orthographic-neighborhood size.

			95% *CI*	
Orthographic neighborhood	Word length (slope)	*SE*	Lower	Upper
1	10	1	7	13
8	13	1	11	16
14	17	2	12	21

Table A4 displays the interaction effect of lexical frequency and orthographic-neighborhood size on first-pass gaze durations per word. Increasing neighborhood size reduced the effect of lexical frequency (high-frequency words were read faster than low-frequency words).

**Table A4.** Gaze duration: Interaction of lexical frequency and orthographic-neighborhood size.

			95% *CI*	
Orthographic neighborhood	Log-frequency (slope)	*SE*	Lower	Upper
1	-24	4	-33	-16
8	-16	4	-24	-9
14	-9	4	-17	0

Table A5 displays the interaction effect of word length and serial text position on first-pass gaze durations per word. The word-length effect (long words took longer to read than short words) decreased as readers progressed through the poems.

**Table A5.** Gaze duration: Interaction of word length and serial text position.

			95% *CI*	
Text position	Word length (slope)	*SE*	Lower	Upper
15	17	2	14	19
36	13	1	11	16
57	10	1	8	13

**Total reading times**

Table A6 displays the interaction effect of word length and orthographic-neighborhood size on total reading times per word. Increasing neighborhood size amplified the word-length effect (long words take longer to read than short words).

**Table A6.** Total reading time per word: Interaction of word length and orthographic-neighborhood size.

			95% *CI*	
Orthographic neighborhood	Word length (slope)	*SE*	Lower	Upper
1	48	6	36	60
8	73	6	62	83
14	97	9	79	116

Table A7 displays the interaction effect of lexical frequency and orthographic-neighborhood size on total reading times per word. Increasing neighborhood size reduced the effect of lexical frequency (high-frequency words were read faster than low-frequency words).

**Table A7.** Total reading time per word: Interaction of lexical frequency and orthographic-neighborhood size.

			95% *CI*	
Orthographic neighborhood	Log-frequency (slope)	*SE*	Lower	Upper
1	-151	12	-174	-128
8	-130	9	-147	-113
14	-110	12	-133	-87

Table A8 displays the interaction effect of word length and serial text position on total reading times per word. The effect of word length (long words take longer to read) decreased as readers progressed through the text.

**Table A8.** Total reading time per word: Interaction of word length and serial text position.

			95% *CI*	
Text position	Word length (slope)	*SE*	Lower	Upper
16	87	6	75	100
37	73	6	62	83
58	58	6	46	70

Table A9 displays the interaction effect of word length and line position on total reading times per word. The effect of word length (long words take longer to read) was more pronounced in non-final positions of the verse line than in line-final positions.

**Table A9.** Total reading time per word: Interaction of word length and line position.

			95% *CI*	
Line position	Word length (slope)	*SE*	Lower	Upper
Non-final	83	5	73	92
Final	63	8	46	79
